# Supplementary material for: Dutch tenants at risk of eviction: Identifying predictors of eviction orders
Source: PLoS One. 2021 Jul 14;16(7):e0254489. doi: 10.1371/journal.pone.0254489 (PMC8279371; doi:10.1371/journal.pone.0254489)
Supplement: S1 File — (PDF) [file pone.0254489.s001.pdf]

Interviewer Code | | |

Respondentnummer | | |

Stadnummer | | |

Risico- en beschermende factoren bij huisuitzetting

Copyright:
UMC St Radboud
Onderzoekscentrum maatschappelijke zorg (Omz)

maart 2012

1 Benadering en non-respons

Interviewer, VUL DEZE PAGINA ZELF IN VOORDAT JE DOORGAAT MET HET INTERVIEW.

[VUL HIER DE CODE IN]

Stad ____(2 cijfers) Interviewer____ (2 cijfers) Respondentnummer ______________(3 cijfers)

Afspraken:

__ __ - __ __ - __ __ __ __	O 0 = Niet aanwezig<br>O 1 = Aanwezig
__ __ - __ __ - __ __ __ __	O 0 = Niet aanwezig<br>O 1 = Aanwezig
__ __ - __ __ - __ __ __ __	O 0 = Niet aanwezig<br>O 1 = Aanwezig

INDIEN ER CONTACT IS MET DE RESPONDENT: GEEF KORT INFORMATIE OVER HET ONDERZOEK

Bent u de hoofdhuurder van uw woning?

O 0 = Ja
O 1 = Nee

Wilt u de toestemmingsverklaring tekenen?

O 0 = De toestemmingsverklaring is door respondent niet ondertekend.
O 1 = De toestemmingsverklaring is door respondent ondertekend.

INDIEN RESPONDENT AANGEEFT NIET MEE TE WILLEN DOEN:
Wat is de reden dat u niet aan het onderzoek wil deelnemen?

➔ *Vul reden in op non-respons formulier!* BEDANK RESPONDENT EN SLUIT AF.

Interviewlocatie:

O 1 = Bij de respondent thuis
O 2 = Ergens anders, namelijk: ____

Geslacht:

O 1 = Man
O 2 = Vrouw

Wat is uw geboortedatum?

__ __ - __ __ - __ __ __ __

Wat zijn de vier cijfers van uw postcode?

__ __ __ __

Vul hier de begintijd van het interview in:

_ _ UU _ _ MM

UMC St Radboud

Respondentnummer | | | |

Verklaring van Toestemming

Risico- en beschermende factoren bij huisuitzetting

Ik werk mee aan het onderzoek naar huurachterstand en huisuitzetting. Het onderzoek wordt uitgevoerd door het Onderzoekscentrum maatschappelijke zorg van het UMC St Radboud in Nijmegen.

Het onderzoek bestaat uit twee delen. In het eerste deel word ik geïnterviewd. Dit interview gaat onder andere over mijn financiële situatie, mijn gezondheidstoestand, mijn sociale contacten en mijn hulpbehoeften. Dit gesprek duurt ongeveer 1 tot 1,5 uur. Na het interview krijg ik € 20 voor mijn medewerking.

In het tweede gedeelte wordt nagegaan of de huisuitzetting wel of niet doorgegaan is en wat mijn verblijfplaats is en wordt mogelijk informatie ingewonnen over hulptrajecten. Deze informatie wordt verkregen op basis van registraties van woningcorporaties, deurwaarders of andere partijen. Door dit formulier te ondertekenen stem ik hiermee in. Door dit formulier te ondertekenen stem ik ermee in dat voornoemde informatie door de gerechtsdeurwaarder aan de woningstichting en/of aan het UMC St Radboud wordt verstrekt, alsmede dat de woningstichting deze informatie aan het UMC St Radboud verstrekt. Ik stem erin toe dat deze toestemmingsverklaring, uitsluitend ten behoeve van het onderzoek, in kopie ter beschikking wordt gesteld aan een betrokken woningcorporatie of deurwaarder.

De onderzoekers mogen ook contact opnemen met mij of met de mensen die ik aan het einde van het interview noem, om de informatie voor het tweede gedeelte van het onderzoek te verkrijgen.

Ik weet dat meedoen vrijwillig is en dat ik op ieder moment kan beslissen om toch niet mee te doen. Ik weet dat het onderzoeksteam mijn gegevens kan inzien. Anderen kunnen mijn gegevens niet inzien. Ik weet dat alle gegevens anoniem en vertrouwelijk behandeld zullen worden.

Ik weet wat het doel is van het interview en waarvoor het wordt gedaan. Ik werk mee onder de voorwaarde dat mijn persoonlijke levenssfeer wordt beschermd. Dit gebeurt volgens de regels die het Universitair Medisch Centrum St Radboud Nijmegen heeft voor het beschermen van privacy van mensen. Als ik niet aan het onderzoek wil meedoen, dan heeft dit geen gevolgen voor mijn situatie.

Deze verklaring geldt alleen voor het huidige onderzoek.

□ De onderzoekers mogen later weer contact met mij opnemen om te vragen of ik mee wil werken aan een vervolginterview.

Naam: □ m □ v

Datum van ondertekening: Plaats:

Handtekening:

Ik verklaar hierbij dat ik deze deelnemer volledig heb geïnformeerd over het genoemde onderzoek.

Naam onderzoeker:

Datum van ondertekening: Plaats:

Handtekening:

2 Inleiding

INTRO:

Het Onderzoekscentrum maatschappelijke zorg van het UMC St Radboud te Nijmegen doet onderzoek naar huurachterstanden en huisuitzettingen. Wij willen graag weten wie de mensen zijn die een huurachterstand opbouwen en hoe deze mensen beter geholpen kunnen worden.

In dit interview worden vragen gesteld over allerlei onderwerpen, zoals familie, gezondheid, werk, vriendschappen en uw behoeften aan hulp. We zijn geïnteresseerd in uw mening, dus er zijn geen goede of foute antwoorden.

Het kan zijn dat sommige vragen wat apart op u overkomen. We interviewen heel veel verschillende mensen en om al die mensen met elkaar te kunnen vergelijken, stel ik u ook de vragen die op u misschien niet van toepassing zijn.

Het gesprek duurt één tot anderhalf uur. De vragenlijst is lang, daarom is het belangrijk dat we niet te veel uitweiden, anders krijgen we de lijst niet af. Als ik aangeef dat we door moeten gaan met de volgende vraag is dat dus niet omdat ik niet geïnteresseerd ben. Als u een pauze wilt nemen, dan doen we dat. Het gesprek kan dan wel iets langer duren.

Alles wat u zegt is anoniem en vertrouwelijk. Niemand komt dus te weten wat u heeft geantwoord. Als u een vraag liever niet beantwoordt, omdat deze te gevoelig ligt of te moeilijk is, dan hoeft dat ook niet. In dat geval gaan we gewoon door met de volgende vraag. Dit geldt voor de hele vragenlijst.

Goed? Dan gaan we nu beginnen.

3 Sociaaldemografische kenmerken

We beginnen met wat vragen over uw achtergrond en opleiding.

1. Wat is het geboorteland van uzelf, uw vader en uw moeder? [WANNEER RESPONDENT NEDERLANDSE ANTILLEN AANGEEFT, VRAAG DAN WELK EILAND.]

		Zelf	Vader	Moeder
1 = Nederland		O 1	O 1	O 1
2 = Suriname		O 2	O 2	O 2
3 = Marokko		O 3	O 3	O 3
4 = Turkije		O 4	O 4	O 4
5 = Ander land, namelijk:				
Zelf:	______________________	O 5		
Vader:	______________________		O 5	
Moeder:	______________________			O 5
6 = Onbekend		O 6	O 6	O 6

2. Wat is uw huidige burgerlijke staat?

O 1 = Gehuwd
O 2 = Geregistreerd partnerschap
O 3 = Gescheiden (hieronder valt ook een ontbonden geregistreerd partnerschap)
O 4 = Weduwe/weduwnaar
O 5 = Ongehuwd [DUS NOOIT GESCHEIDEN]

3. Heeft u (stief/pleeg)kinderen? Welke leeftijd hebben uw (stief/pleeg)kinderen?

O 0 = Nee
☐ 1 = Ja, __ kinderen jonger dan 18 jaar, __ kinderen van 18 jaar of ouder.
☐ 2 = Ja, __ stiefkinderen jonger dan 18 jaar, __ stiefkinderen van 18 jaar of ouder.
☐ 3 = Ja, __ pleegkinderen jonger dan 18 jaar, __ pleegkinderen van 18 jaar of ouder.

4. Wat is uw huidige leefsituatie?

O 1 = Alleenstaand
O 2 = Met partner
O 3 = Met partner en kind(eren)
O 4 = Eenoudergezin (alleenstaand met kind(eren))
O 5 = Met ouders
O 6 = Met anderen (geen partnerrelatie, wel gezamenlijk huishouden)

5. Uit hoeveel mensen (volwassenen en kinderen) bestaat uw huishouden (inclusief uzelf)?

__ __

6. Wat is de hoogste opleiding die u met een diploma heeft afgesloten? [OPEN]
[BIJ UITBLIJVEN VAN EEN ANTWOORD VRAAG DAN: *bent u naar de basisschool geweest en heeft u dit afgemaakt, bent u naar de middelbare school geweest en heeft u dit afgemaakt* enz.]
[INDIEN IN BUITENLAND OPGELEID, DAN VERGELIJKBARE NIVEAU AANGEVEN.]

	Hoogst voltooide opleiding
Geen opleiding (lager onderwijs / basisschool: niet afgemaakt)	O 1
Lager onderwijs (speciaal onderwijs (LOM, ZMOK, BLO, MLK, ZMLK), gewone lagere school / basisschool)	O 2
Lager of voorbereidend beroepsonderwijs (zoals LTS, LEAO, LHNO, VMBO)	O 3
Middelbaar algemeen voortgezet onderwijs (zoals MAVO, (M)ULO, MBO-kort (niveau 1 of 2), VMBO-TL)	O 4
Middelbaar beroepsonderwijs en beroepsbegeleidend onderwijs (zoals MBO-lang (niveau 3 of 4), MTS, MEAO, BOL, BBL, INAS)	O 5
Hoger algemeen en voorbereidend wetenschappelijk onderwijs (zoals HAVO, VWO, atheneum, gymnasium, HBS, MMS)	O 6
Hoger beroepsonderwijs (zoals HBO, HTS, HEAO, HBO-V, kandidaats wetenschappelijk onderwijs)	O 7
Wetenschappelijk onderwijs (universiteit)	O 8
Anders, namelijk: ____	O 9

7. Kunt u aangeven of u moeite heeft met de Nederlandse taal?

	Geen moeite	Een beetje moeite	Veel moeite
a. Heeft u moeite met het spreken van Nederlands?	O 1	O 2	O 3
b. Heeft u moeite met het lezen van Nederlands, bijvoorbeeld bij het lezen van kranten, brieven of folders?	O 1	O 2	O 3
c. Heeft u moeite met het schrijven van Nederlands?	O 1	O 2	O 3
d. Heeft u moeite een gesprek in het Nederlands te volgen?	O 1	O 2	O 3

4 Algemeen welbevinden

We gaan nu verder met een vraag over uw leven in het algemeen.

8. Hoe ervaart u uw leven in het algemeen?
[VRAAG EVENTUEEL: "Mijn leven in het algemeen vind ik: …"] [GEBRUIK KAART 1]

- O 1 = Vreselijk
- O 2 = Slecht
- O 3 = Matig
- O 4 = Niet slecht/niet goed
- O 5 = Redelijk
- O 6 = Goed
- O 7 = Prima

5 Huisvesting

Dan gaan we nu door met vragen over uw huisvesting.

5.1 Huisvesting

9. Sinds wanneer woont u in uw huidige woning? [DAG-MAAND-JAAR]

__ __ - __ __ - __ __ __ __

De volgende vragen gaan over de plek waar u <u>op dit moment</u> woont. [GEBRUIK KAART 1]

	Vreselijk	Slecht	Matig	Niet slecht/ niet goed	Redelijk	Goed	Prima
**10. Wat vindt u van de omstandigheden waarin u leeft?**	O1	O2	O3	O4	O5	O6	O7
**11. Wat vindt u van de privacy die u hier heeft?**	O1	O2	O3	O4	O5	O6	O7
**12. Als het zou kunnen, hoe zou u het dan vinden om voor langere tijd op deze plek te blijven wonen?**	O1	O2	O3	O4	O5	O6	O7

13. Bent u in het verleden ooit dakloos geweest?

- O 0 = Nee → GA NAAR VRAAG 16
- O 1 = Ja

14. In welk jaar was u voor de eerste keer dakloos?

__ __ __ __

15. Hoe lang bent in totaal ongeveer dakloos geweest in uw leven?

- O ………..jaar
- O ………..maanden
- O ………..weken
- O ………..dagen

5.2 Zorgbehoeften huisvesting, huishouden en zelfverzorging

16. Ik wil nu graag van u weten of u op de gebieden die ik zo noem op dit moment hulp wilt en hulp krijgt:
A: Wilt u hulp op dit gebied?
B: Krijgt u hierbij hulp van instanties?

	**Wil hulp?**		**Krijgt hulp?**	
	Nee	Ja	Nee	Ja
**a. Huisvesting** *(Zoeken en houden van woonruimte, opknappen en inrichten van woonruimte)*	O 0	O 1	O 0	O 1
**b. Huishoudelijke taken en zelfverzorging (persoonlijke verzorging)** *(Boodschappen doen, maaltijd bereiden, was doen, schoonmaken van woonruimte, kleden, tanden poetsen, in en uit bed gaan, naar toilet (kunnen) gaan)*	O 0	O 1	O 0	O 1

6 Dagbesteding, werk en vrije tijd

De volgende vragen gaan over uw dagbesteding en vrije tijd. We beginnen met dagbesteding.

6.1 Dagbesteding

17. Hoe vult u uw dag in? Ik loop meerdere opties langs. Vult u uw dag in met (een)…?

a. Reguliere betaalde baan, met arbeidscontract	O 0 Nee	O 1 Ja
b. Betaalde baan in sociale werkvoorziening	O 0 Nee	O 1 Ja
c. Baan in kader van arbeidsintegratieproject	O 0 Nee	O 1 Ja
d. Werkervaringsplaats	O 0 Nee	O 1 Ja
e. Vrijwilligerswerk	O 0 Nee	O 1 Ja
f. Opleiding, studie	O 0 Nee	O 1 Ja
g. Dagactiviteitencentrum	O 0 Nee	O 1 Ja
h. Huishouden/verzorging van kinderen	O 0 Nee	O 1 Ja
i. Anders, namelijk …………………………………………………………………………………..	O 0 Nee	O 1 Ja

6.2 Werk en vrije tijd

Dan wil ik u nu graag wat vragen stellen over uw werk en uw vrije tijd. [GEBRUIK KAART 1]

		Vreselijk	Slecht	Matig	Niet slecht/ niet goed	Redelijk	Goed	Prima
**18. Wat vindt u van uw werk?**	O Werkt niet	O1	O2	O3	O4	O5	O6	O7
**19. Wat vindt u van uw werkplek (de fysieke omgeving)?**	O Werkt niet	O1	O2	O3	O4	O5	O6	O7
**20. Wat vindt u van de hoogte van het inkomen dat u met dit werk verdient?**	O Werkt niet	O1	O2	O3	O4	O5	O6	O7
**21. Wat vindt u van de manier waarop u uw vrije tijd besteedt?**		O1	O2	O3	O4	O5	O6	O7
**22. Wat vindt u van de mogelijkheden die u heeft om te genieten van fijne of mooie dingen?**		O1	O2	O3	O4	O5	O6	O7
**23. Wat vindt u van de hoeveelheid lol die u heeft?**		O1	O2	O3	O4	O5	O6	O7
**24. Wat vindt u van de hoeveelheid ontspanning in uw leven?**		O1	O2	O3	O4	O5	O6	O7

6.3 Zorgbehoeften dagbesteding en dagritme

25. Ik wil nu graag van u weten of u op de gebieden die ik zo noem op dit moment hulp wilt en hulp krijgt:
A: Wilt u hulp op dit gebied?
B: Krijgt u hierbij hulp van instanties?

	**Wil hulp?**		**Krijgt hulp?**	
	Nee	Ja	Nee	Ja
**a. Het vinden van (betaald) werk**	O 0	O 1	O 0	O 1
**b. Dagbesteding en vrije tijd** *(Werken aan een beter dag-/nachtritme, aan clubs of cursussen meedoen, uitgaan, naar winkels, bibliotheek, etc.)*	O 0	O 1	O 0	O 1

7 Financiën

Ik zou nu graag wat verder in willen gaan op uw financiële situatie.

7.1 Financiën

Eerst zal ik u een aantal vragen stellen over uw financiële situatie in het algemeen.

26. Wie beheert de financiën in uw huishouden?

- ○1 = Ikzelf
- ○2 = Mijn echtgenoot/partner
- ○3 = Mijn zoon/dochter [INCLUSIEF STIEFKINDEREN]
- ○4 = Vader/moeder (van mijzelf/mijn partner)
- ○5 = Iemand anders, namelijk ______________________________________

27. Had uw huishouden in het afgelopen jaar inkomsten uit de volgende bronnen?
[HET GAAT HIER OM DE INKOMSTENBRONNEN VAN ALLE LEDEN VAN HET HUISHOUDEN]

a.	Loon, wit werk	○ 0 Nee	○ 1 Ja
b.	Loon, zwart werk	○ 0 Nee	○ 1 Ja
c.	Uitkering ziekte of arbeidsongeschiktheid (WAO, AAW, ziektewet, WIA, Wajong)	○ 0 Nee	○ 1 Ja
d.	Uitkering wegens pensionering (AOW, pensioen)	○ 0 Nee	○ 1 Ja
e.	Bijstandsuitkering (RWW, AWB, WWB, WIJ)	○ 0 Nee	○ 1 Ja
f.	Uitkering werkloosheid (WW)	○ 0 Nee	○ 1 Ja
g.	Huurtoeslag	○ 0 Nee	○ 1 Ja
h.	Zorgtoeslag	○ 0 Nee	○ 1 Ja
i.	Kindgebonden budget, kinderbijslag, kinderopvangtoeslag	○ 0 Nee	○ 1 Ja
j.	Tegemoetkoming schoolkosten [voor kinderen in MBO, VAVO en particulier onderwijs]	○ 0 Nee	○ 1 Ja
k.	Belastingteruggaaf / heffingskortingen	○ 0 Nee	○ 1 Ja
l.	Alimentatie en bijdragen voor kinderen	○ 0 Nee	○ 1 Ja
m.	Inkomsten van partner / ouders, incl. zakgeld	○ 0 Nee	○ 1 Ja
n.	Studiefinanciering, beurs	○ 0 Nee	○ 1 Ja
o.	Jeugdwerkgarantieplan	○ 0 Nee	○ 1 Ja
p.	Giften	○ 0 Nee	○ 1 Ja
q.	Prostitutie	○ 0 Nee	○ 1 Ja
r.	Bedelen	○ 0 Nee	○ 1 Ja
s.	Illegale activiteiten (dealen, diefstal, beroving, heling)	○ 0 Nee	○ 1 Ja
t.	Overige inkomsten, namelijk: ___________________	○ 0 Nee	○ 1 Ja

28. Krijgt u hulp bij het beheren van uw financiën?

- ○ 0 = Nee
- ○ 1 = Ja, van een instantie, namelijk: ____
- ○ 2 = Ja, van vrienden/familie
- ○ 3 = Ja, anders, namelijk: ____

29. Staat u onder bewind van een bewindvoerder?

- ○ 0 = Nee
- ○ 1 = Ja

30. Is er loonbeslag gelegd op uw inkomen en/of op het inkomen van uw partner?

○ 0 = Nee
○ 1 = Ja, op mijn inkomen
○ 2 = Ja, op het inkomen van mijn partner
○ 3 = Ja, op mijn inkomen en dat van mijn partner

31. [VERWIJS NAAR VRAAG 27] **Hoeveel ontving uw hele huishouden de afgelopen maand vanuit al deze bronnen gezamenlijk (netto)?**

€ ______________________ ○ Weet niet

32. [ALS ER SPRAKE IS VAN BEWINDVOERING, LOONBESLAG EN/OF HULP BIJ BEHEREN VAN FINANCIËN] **Hoeveel geld ontving uw hele huishouden de afgelopen maand daadwerkelijk om te besteden?**

€ ______________________ ○ Weet niet

33. Wat waren voor u in de afgelopen 6 maanden de belangrijkste dingen om geld aan uit te geven? Noemt u maar het eerste dat in u opkomt, de volgorde maakt niet uit. [MAXIMAAL 5, MAAR MINDER MAG OOK]
En kunt u aangeven welke van deze dingen voor u het belangrijkst was (1), welke iets minder belangrijk (2), enzovoorts?

Belangrijkste uitgaven:	Rangorde (1 = belangrijkst, 5 = minst belangrijk)
______________________	☐
______________________	☐
______________________	☐
______________________	☐
______________________	☐

34. Gemiddeld genomen, hoeveel geld had u de afgelopen maand om voor uzelf uit te geven als u de huur of maaltijden niet meerekent?
[HET GAAT HIER OM WAT DE RESPONDENT VOOR ZICHZELF OVERHOUDT, ALS ALLE VASTE LASTEN BETAALD ZIJN.]

€ ______________________ ○ Weet niet

[GEBRUIK KAART 1]

	Vreselijk	Slecht	Matig	Niet slecht/ niet goed	Redelijk	Goed	Prima
**35. Wat vindt u van de hoeveelheid geld die u ontvangt?**	O1	O2	O3	O4	O5	O6	O7
**36. Wat vindt u van de hoeveelheid geld die u achter de hand heeft?**	O1	O2	O3	O4	O5	O6	O7
**37. Wat vindt u van de hoeveelheid geld die u ter beschikking heeft om uit te geven aan leuke dingen?**	O1	O2	O3	O4	O5	O6	O7

7.2 Financiële vaardigheden

Nu volgen enkele vragen over omgaan met geld en de vaardigheden die u heeft op het gebied van het beheren van uw financiën.

38. Wilt u bij de volgende handelingen en vaardigheden aangeven of u dit nooit, zelden, soms, vaak of altijd doet? [GEBRUIK KAART 2]

		Nooit	Zelden	Soms	Vaak	Altijd
a	Opent u dagelijks de post?	O 1	O 2	O 3	O 4	O 5
b	Reageert u op brieven als dat nodig is?	O 1	O 2	O 3	O 4	O 5
c	Bergt u de financiële post op, bijvoorbeeld in een map/klapper?	O 1	O 2	O 3	O 4	O 5
d	Bent u in staat uw financiën zelfstandig te beheren? *(administratie bijhouden, budgetteren, rondkomen met geld)*	O 1	O 2	O 3	O 4	O 5
e	Komt u uit met uw geld?	O 1	O 2	O 3	O 4	O 5
f	Spaart u?	O 1	O 2	O 3	O 4	O 5
g	Lukt het u om reclames/aanbiedingen te weerstaan?	O 1	O 2	O 3	O 4	O 5
h	Koopt u regelmatig iets als troost?	O 1	O 2	O 3	O 4	O 5
i	Koopt u regelmatig iets om mee te doen met anderen / erbij te horen?	O 1	O 2	O 3	O 4	O 5
j	Koopt u regelmatig iets omdat u de kinderen niet tekort wilt doen? O Geen kinderen	O 1	O 2	O 3	O 4	O 5
k	Neemt u zelf contact op met schuldeisers?	O 1	O 2	O 3	O 4	O 5

7.3 Zorgbehoeften financiën

39. Ik wil nu graag van u weten of u op het gebied dat ik zo noem <u>op dit moment</u> hulp wilt en hulp krijgt:
A: Wilt u hulp op dit gebied?
B: Krijgt u hierbij hulp van instanties?

	**Wil hulp?**		**Krijgt hulp?**	
	Nee	Ja	Nee	Ja
**a. Financiën** *(Krijgen van inkomsten, beheer geld, leren omgaan met geld, invullen formulieren, administratie)*	○ 0	○ 1	○ 0	○ 1

7.4 Schulden

40. Heeft u op dit moment schulden? *(Bijv. huurschuld, schulden bij gas, water en/of licht, schulden bij ziektekostenverzekeraar of andere verzekeringsmaatschappij, lening bij bank/ kredietbank/ giro, lening of schuld bij familie of vrienden, schulden bij postorderbedrijven, schulden bij de belasting maar GEEN studie- of hypotheekschuld)*

○ 0 = Nee → **Had u een maand geleden schulden?** ○ 0 Nee ○ 1 Ja
○ 1 = Ja

[ALS RESPONDENT AANGEEFT DAT ZIJN/HAAR SCHULDEN RECENTELIJK ZIJN AFBETAALD, STEL DAN DE REST VAN DE VRAGEN VAN DEZE SECTIE EN DE SECTIES “HUURACHTERSTAND” EN “COPING” OVER DE SITUATIE VAN EEN MAAND GELEDEN!]

41. Waar heeft u op dit moment schulden? [MEERDERE ANTWOORDEN MOGELIJK]

☐ 1 = Huurschuld
☐ 2 = Schuld bij leverancier van gas/water/elektriciteit
☐ 3 = Verzekeringsschuld
☐ 4 = Persoonlijke lening bij bank
☐ 5 = Belastingschuld
☐ 6 = Schuld bij postorderbedrijven
☐ 7 = Lening of schuld bij familie of vrienden
☐ 8 = Anders, namelijk: ____

42. Hoe hoog is het totale schuldbedrag nu ongeveer? [ROND AF OP HELE EURO’S.]

€ ________________________ ○ Weet niet

43. Hoe hoog is uw huurschuld nu ongeveer? [ROND AF OP HELE EURO’S. ALS RESPONDENT HET BEDRAG NIET WEET, VRAAG DAN HOEVEEL MAANDEN ACHTERSTAND HIJ/ZIJ HEEFT.]

€ ________________________ ○ Weet niet

_______ maanden

7.5 Huurachterstand

Nu zou ik u graag wat meer vragen willen stellen over uw huur en huurschuld.
[ALS DE RESPONDENT OP DIT MOMENT GEEN HUURSCHULD MEER HEEFT, STEL DAN DE VRAGEN IN DEZE SECTIE OVER DE SITUATIE VAN EEN MAAND GELEDEN]

44. Op welke wijze betaalt u normaal gesproken uw huur?

O 1 = Via automatische incasso
O 2 = Via een acceptgiro
O 3 = Met een eigen overschrijving
O 4 = Pinnen/contant op het kantoor van de woningcorporatie
O 5 = Anders, namelijk: ____

45. Hoe hoog is uw totale maandelijkse huur? En weet u hoeveel de kale huur is? [ROND AF OP HELE EURO'S.]

Totale huur: € ______________ O Weet niet

Kale huur: € ______________ O Weet niet

46. U gaf aan dat u een huurachterstand heeft. Wie of wat heeft volgens u het meest bijgedragen aan het ontstaan van deze situatie?

O 1 = Ikzelf
O 2 = Eén of meer van mijn gezinsleden
O 3 = Andere mensen uit mijn omgeving
O 4 = Instanties
O 5 = Anders, namelijk: ____

47. Heeft u het gevoel dat deze huurachterstand en daarmee de dreiging van huisuitzetting voorkomen had kunnen worden?

O 0 = Nee
O 1 = Ja

48. Kunt u dat toelichten? [ZO VOLLEDIG MOGELIJK OPSCHRIJVEN]

………

………

………

………

………

………

………

………

49. De volgende vragen gaan over uw gevoelens bij deze situatie. Wilt u bij iedere vraag aangeven in hoeverre dit voor u opgaat? [GEBRUIK KAART 3]

	Helemaal niet	Grotendeels niet	Enigszins niet	Neutraal	Enigszins wel	Grotendeels wel	Helemaal wel
a. Maakt de situatie u boos?	O 1	O 2	O 3	O 4	O 5	O 6	O 7
b. Geeft de situatie u gevoelens van stress?	O 1	O 2	O 3	O 4	O 5	O 6	O 7
c. Slaapt u slechter door deze situatie?	O 1	O 2	O 3	O 4	O 5	O 6	O 7
d. Maakt de situatie u verdrietig?	O 1	O 2	O 3	O 4	O 5	O 6	O 7
e. Voelt u zich machteloos door deze situatie?	O 1	O 2	O 3	O 4	O 5	O 6	O 7
f. Schaamt u zich omdat u in deze situatie zit?	O 1	O 2	O 3	O 4	O 5	O 6	O 7
g. Vindt u het eerlijk dat u in deze situatie zit?	O 1	O 2	O 3	O 4	O 5	O 6	O 7
h. Vindt u het terecht dat u in deze situatie zit?	O 1	O 2	O 3	O 4	O 5	O 6	O 7

50. Heeft u een dagvaarding ontvangen waarin u wordt opgeroepen voor een zitting bij de rechtbank vanwege deze huurachterstand?

O 0 = Nee
O 1 = Ja
O 2 = Weet ik niet

51. Heeft de zitting bij de kantonrechter inmiddels plaatsgevonden?

O 0 = Nee → GA NAAR VRAAG 55
O 1 = Ja
O 2 = Weet ik niet → GA NAAR VRAAG 55

52. Was u of iemand anders uit uw huishouden aanwezig op deze zitting?

O 0 = Nee
O 1 = Ja → **Wie was aanwezig?**
  O 1 = Ik → GA NAAR VRAAG 54
  O 2 = Iemand anders, namelijk: ________________ ________________

53. Waarom was u niet aanwezig op de zitting? [MEERDERE ANTWOORDEN MOGELIJK]

☐ 1 = Geen tijd
☐ 2 = Ziekte
☐ 3 = Vond ik niet nodig
☐ 4 = Anders, namelijk: ______________________________

54. Is er al een uitspraak? Zo ja, wat was de uitspraak?

O 0 = Nog geen uitspraak
O 1 = Ja, betaalvonnis
O 2 = Ja, ontruimingsvonnis → ontruimingsdatum: ________________
O 3 = Ja, geen betaal- of ontruimingsvonnis
O 4 = Anders, namelijk: ______________________________
O 5 = Weet ik niet

55. Heeft u sinds het ontstaan van uw huurachterstand hulp gekregen (bijvoorbeeld van schuldhulpverlening, de woningcorporatie of maatschappelijk werk) om u te helpen het probleem op te lossen? En zo ja, wanneer was dat? [MEERDERE ANTWOORDEN MOGELIJK]

☐ 0 = Nee → GA NAAR VRAAG 57
☐ 1 = Ja, nog voordat de deurwaarder ingeschakeld werd
☐ 2 = Ja, in de periode dat de deurwaarder de zaak in behandeling had
☐ 3 = Ja, na de uitspraak van de rechter

56. Van wie heeft u hulp gekregen? [MEERDERE ANTWOORDEN MOGELIJK]

☐ 1 = Schuldhulpverlening
☐ 2 = Woningcorporatie
☐ 3 = Maatschappelijk werk
☐ 4 = Anders, namelijk: ____

57. Bent u al eens eerder gedagvaard vanwege een huurachterstand, vanwege overlast of vanwege andere zaken die kunnen leiden tot huisuitzetting (bv. illegale onderverhuur, hennepteelt)?
[MEERDERE ANTWOORDEN MOGELIJK. HET GAAT HIER NIET ALLEEN OM DE HUIDIGE WOONRUIMTE, MAAR OOK OM VORIGE WONINGEN. HUIDIGE DAGVAARDING TELT NIET MEE.]

☐ 0 = Nee
☐ 1 = Ja, vanwege een huurachterstand → ___keer
☐ 2 = Ja, vanwege overlast → ___keer
☐ 3 = Ja, vanwege andere redenen → ___keer, namelijk: ____________________

58. Bent u in het verleden uw huis uitgezet vanwege een huurachterstand, vanwege overlast, of vanwege andere redenen? [MEERDERE ANTWOORDEN MOGELIJK]

☐ 0 = Nee
☐ 1 = Ja, vanwege een huurachterstand → ___keer
☐ 2 = Ja, vanwege overlast → ___keer
☐ 3 = Ja, vanwege andere redenen → ___keer, namelijk: ____________________

7.6 Coping: omgaan met een dreigende huisuitzetting

59. We willen graag weten hoe mensen omgaan met problemen en tegenslagen in hun leven. Iedereen doet dat anders. Ik noem zodadelijk een aantal manieren op en ik wil u vragen om aan te geven in hoeverre dat een manier is waarop u met uw huidige situatie omgaat. Er zijn geen goede of foute antwoorden. Sommige vragen lijken misschien wat op elkaar, maar ik wil u vragen toch alle vragen te beantwoorden. De vraag is dus of u aan kunt geven hoe u omgaat met uw situatie: de huurachterstand en eventuele dreigende huisuitzetting. U kunt dat doen door één van deze [WIJS OP KAART 4] **antwoorden te geven.**

		Helemaal niet	Een beetje	Helemaal wel
a.	Uw hart luchten bij een vriend(in).	O 1	O 2	O 3
b.	De zaken om u heen zodanig herorganiseren dat uw probleem de grootste kans heeft opgelost te worden.	O 1	O 2	O 3
c.	Over alle mogelijke oplossingen nadenken voordat u besluit wat te doen.	O 1	O 2	O 3
d.	Proberen uzelf af te leiden van het probleem.	O 1	O 2	O 3
e.	Medeleven en begrip van iemand accepteren.	O 1	O 2	O 3
f.	Uw uiterste best doen om te zorgen dat niemand ziet hoe slecht uw situatie eigenlijk is.	O 1	O 2	O 3

		Helemaal niet	Een beetje	Helemaal wel
g.	Met mensen praten over de situatie, omdat erover praten u helpt om u beter te voelen.	○ 1	○ 2	○ 3
h.	Bepaalde doelen voor uzelf stellen om met de situatie om te kunnen gaan.	○ 1	○ 2	○ 3
i.	Al uw opties zeer zorgvuldig tegen elkaar afwegen.	○ 1	○ 2	○ 3
j.	Dagdromen over betere tijden.	○ 1	○ 2	○ 3
k.	Het probleem op verschillende manieren proberen op te lossen totdat u een manier vindt die werkt.	○ 1	○ 2	○ 3
l.	Uw angsten en zorgen toevertrouwen aan een vriend(in).	○ 1	○ 2	○ 3
m.	Meer tijd alleen doorbrengen dan gewoonlijk.	○ 1	○ 2	○ 3
n.	Mensen over uw situatie vertellen omdat erover praten u helpt om oplossingen te bedenken.	○ 1	○ 2	○ 3
o.	Nadenken over wat er gedaan zou moeten worden om de zaken op te lossen.	○ 1	○ 2	○ 3
p.	Uw volledige aandacht richten op het oplossen van het probleem.	○ 1	○ 2	○ 3
q.	In gedachten een plan van aanpak maken.	○ 1	○ 2	○ 3
r.	Meer televisie kijken dan gewoonlijk.	○ 1	○ 2	○ 3
s.	Naar iemand toegaan (vriend(in) of hulpverlener) om u te helpen u beter te voelen.	○ 1	○ 2	○ 3
t.	Voet bij stuk houden en vechten voor wat u wilt in deze situatie.	○ 1	○ 2	○ 3
u.	Vermijden om onder de mensen te zijn.	○ 1	○ 2	○ 3
v.	Uzelf verliezen in een hobby of sport om het probleem te vermijden.	○ 1	○ 2	○ 3
w.	Naar een vriend(in) gaan om u te helpen u beter te voelen over het probleem.	○ 1	○ 2	○ 3
x.	Naar een vriend(in) gaan voor advies over hoe u de situatie kunt veranderen.	○ 1	○ 2	○ 3
y.	Medeleven en begrip accepteren van vrienden met hetzelfde probleem.	○ 1	○ 2	○ 3
z.	Meer slapen dan gewoonlijk.	○ 1	○ 2	○ 3
aa.	Fantaseren over hoe de zaken anders hadden kunnen zijn.	○ 1	○ 2	○ 3
bb.	Uzelf identificeren met karakters in romans en films.	○ 1	○ 2	○ 3
cc.	Proberen het probleem op te lossen.	○ 1	○ 2	○ 3
dd.	Wensen dat mensen u gewoon met rust zouden laten.	○ 1	○ 2	○ 3
ee.	Hulp van een vriend(in) of familielid accepteren.	○ 1	○ 2	○ 3
ff.	Geruststelling zoeken bij degenen die u het beste kennen.	○ 1	○ 2	○ 3
gg.	Proberen om zorgvuldig uw koers te bepalen in plaats van impulsief te handelen.	○ 1	○ 2	○ 3

60. Hoe waarschijnlijk vindt u het dat u in het komende half jaar uit uw huis gezet wordt? [GEBRUIK KAART 5]

○ 1 = Zeer onwaarschijnlijk
○ 2 = Onwaarschijnlijk
○ 3 = Niet onwaarschijnlijk/niet waarschijnlijk
○ 4 = Waarschijnlijk
○ 5 = Zeer waarschijnlijk

61. Als u daadwerkelijk uit uw huis wordt gezet, weet u dan waar u naartoe kunt gaan?

O 1 = Nee
O 2 = Ja, namelijk: ____

8 Sociale relaties

8.1 Sociale contacten

We gaan nu verder met vragen over uw sociale relaties. We beginnen met een vraag over uw jeugd en daarna stel ik u een paar vragen over uw familie en andere sociale contacten.
[GEBRUIK KAART 1]

	Vreselijk	Slecht	Matig	Niet slecht/ niet goed	Redelijk	Goed	Prima
**62. Hoe was de financiële situatie in uw gezin toen u nog een kind was?**	O1	O2	O3	O4	O5	O6	O7
**63. Wat vindt u van de manier waarop u en uw familie met elkaar omgaan?**	O1	O2	O3	O4	O5	O6	O7
**64. Wat vindt u van de manier waarop dingen in het algemeen verlopen tussen u en uw familie?**	O1	O2	O3	O4	O5	O6	O7
**65. Wat vindt u van de dingen die u met anderen, exclusief familie, doet?**	O1	O2	O3	O4	O5	O6	O7
**66. Wat vindt u van de hoeveelheid tijd die u met anderen, exclusief familie, doorbrengt?**	O1	O2	O3	O4	O5	O6	O7
**67. Wat vindt u van de vrienden en kennissen, die u voor de gezelligheid ziet?**	O1	O2	O3	O4	O5	O6	O7

8.2 Zorgbehoeften Sociale relaties

68. Ik wil nu graag weer van u weten of u op de gebieden die ik zo noem <u>op dit moment</u> hulp wilt en hulp krijgt:
A: Wilt u hulp op dit gebied?
B: Krijgt u hierbij hulp van instanties?

		**Wil hulp?**		**Krijgt hulp?**	
		Nee	Ja	Nee	Ja
**a. Relatie met familie**<br>*(Onderhouden relaties met familie, werken aan herstel van familierelaties, omgaan met conflicten)*		O 0	O 1	O 0	O 1
**b. Sociale contacten (vrienden/kennissen)**<br>*(Contacten leggen en behouden, samen dingen doen, omgaan met conflicten, intieme relatie aangaan)*		O 0	O 1	O 0	O 1
**c. Relatie met uw partner**<br>*(Samen dingen doen, omgaan met conflicten, seksualiteit, intieme relatie aangaan)*	O Heeft geen partner	O 0	O 1	O 0	O 1

		Wil hulp?		Krijgt hulp?	
		Nee	Ja	Nee	Ja
**d. Kinderen: hulp in verband met contactleggen met kinderen of hulp voor de kinderen** *(Begeleiding bij het leggen van contact met de kinderen/bezoekregeling, doorverwijzing naar andere instanties, advies hoe om te gaan met kinderen, kinderen steun geven)*	O Heeft geen kinderen	O 0	O 1	O 0	O 1

8.3 Vervulling basisbehoeften

Dan wil ik u nu graag wat vragen stellen over uzelf en de mensen om u heen.

69. Ik lees u nu een aantal stellingen voor. Wilt u aangeven in hoeverre deze voor u en uw leven opgaan? U kunt dat doen door deze [WIJS OP KAART 6] **antwoordschaal te gebruiken.**

	Helemaal niet waar	Niet waar	Een beetje niet waar	Niet waar en niet onwaar	Een beetje waar	Waar	Helemaal wel waar
a. Ik heb het gevoel dat ik vrij ben om zelf te bepalen hoe ik mijn leven wil leiden.	O1	O2	O3	O4	O5	O6	O7
b. Ik vind de mensen met wie ik omga erg aardig.	O1	O2	O3	O4	O5	O6	O7
c. Vaak voel ik me niet zo competent.	O1	O2	O3	O4	O5	O6	O7
d. Ik heb het gevoel dat ik onder druk sta in mijn leven.	O1	O2	O3	O4	O5	O6	O7
e. Mensen die ik ken vertellen me dat ik goed ben in wat ik doe.	O1	O2	O3	O4	O5	O6	O7
f. Ik kan goed opschieten met de mensen met wie ik in contact kom.	O1	O2	O3	O4	O5	O6	O7
g. Ik ben nogal op mezelf, en heb niet veel sociale contacten.	O1	O2	O3	O4	O5	O6	O7
h. Meestal voel ik me vrij om mijn ideeën en mening te uiten.	O1	O2	O3	O4	O5	O6	O7
i. Ik zie de mensen met wie ik regelmatig omga als mijn vrienden.	O1	O2	O3	O4	O5	O6	O7
j. Ik heb de afgelopen tijd interessante nieuwe vaardigheden kunnen leren.	O1	O2	O3	O4	O5	O6	O7
k. In het dagelijks leven moet ik vaak doen wat mij wordt opgedragen.	O1	O2	O3	O4	O5	O6	O7
l. Mensen in mijn leven geven om mij.	O1	O2	O3	O4	O5	O6	O7
m Op de meeste dagen heb ik een gevoel van voldoening bij wat ik doe.	O1	O2	O3	O4	O5	O6	O7
n. De mensen met wie ik dagelijks contact heb houden meestal rekening met mijn gevoelens.	O1	O2	O3	O4	O5	O6	O7
o. Ik krijg in mijn leven niet echt een kans om te laten zien wat ik kan.	O1	O2	O3	O4	O5	O6	O7
p. Er zijn niet veel mensen met wie ik een hechte band heb.	O1	O2	O3	O4	O5	O6	O7

	Helemaal niet waar	Niet waar	Een beetje niet waar	Niet waar en niet onwaar	Een beetje waar	Waar	Helemaal wel waar
q. Ik heb het gevoel dat ik in dagelijkse situaties redelijk mezelf kan zijn.	O1	O2	O3	O4	O5	O6	O7
r. De mensen met wie ik regelmatig contact heb lijken mij niet erg aardig te vinden.	O1	O2	O3	O4	O5	O6	O7
s. Ik voel me vaak niet capabel.	O1	O2	O3	O4	O5	O6	O7
t. Er zijn weinig mogelijkheden om voor mijzelf te bepalen hoe ik dingen in mijn leven doe.	O1	O2	O3	O4	O5	O6	O7
u. Mensen zijn meestal best aardig tegen mij.	O1	O2	O3	O4	O5	O6	O7

8.4 Sociale steun

Ik ga u nu wat vragen stellen over uw sociale netwerk en sociale steun.

[GEEF RESPONDENT DE BIJLAGE “SOCIALE STEUN” EN HELP HEM/HAAR MET INVULLEN. ZET RESPONDENTNUMMER OP DE BIJLAGE.]

9 Gezondheid

9.1 Lichamelijke gezondheid

Nu zou ik u iets over uw gezondheid willen vragen.

70. Wat vindt u van uw fysieke gesteldheid, conditie? [GEBRUIK KAART 1]

O 1 = Vreselijk
O 2 = Slecht
O 3 = Matig
O 4 = Niet slecht/niet goed
O 5 = Redelijk
O 6 = Goed
O 7 = Prima

71. Heeft u momenteel lichamelijke klachten?

O 0 = Nee → GA NAAR VRAAG 73
O 1 = Ja

72. Kunt u aangeven in welke mate u door lichamelijke klachten belemmerd wordt?

	**In welke mate wordt u door lichamelijke klachten belemmerd …**	Niet belemmerd	Licht belemmerd	Sterk belemmerd
a.	… bij het uitvoeren van dagelijkse bezigheden thuis?	O 1	O 2	O 3
b.	… in uw vrijetijdsbesteding, bijvoorbeeld bij het sporten of het reizen?	O 1	O 2	O 3
c.	… bij het uitvoeren van dagelijkse bezigheden op school of op het werk?	O 1	O 2	O 3

73. Bent u onder behandeling van een arts of specialist?

○ 0 = Nee
○ 1 = Ja

74. Gebruikt u medicijnen voor (uw) lichamelijke klachten?

○ 0 = Nee
○ 1 = Ja

75. Heeft u aanpassingen, extra zorg of toezicht nodig vanwege lichamelijke klachten?

○ 0 = Nee
○ 1 = Ja

76. Bent u tegen ziektekosten verzekerd?

○ 0 = Nee
○ 1 = Ja, basis
○ 2 = Ja, basis + aanvullend
○ 3 = Ja, basis + tandarts
○ 4 = Ja, basis + aanvullend + tandarts
○ 5 = Ja, maar weet niet hoe
○ 6 = Weet niet

9.2 Verslaving

Nu volgen er vragen over alcohol- en druggebruik.

77. Ik wil graag van verschillende middelen van u weten of u ze ooit gebruikt heeft en hoeveel u ze gebruikt heeft.
A. Heeft u ooit … gebruikt? [INDIEN NOOIT GEBRUIKT, DAN IS JAREN OOIT 88 EN AANTAL DAGEN 00]
B. Hoeveel jaren heeft u … gebruikt? [AFRONDEN OP HELE JAREN. LET OP! HIER GELDT DAT HET MIDDEL **MINIMAAL 3 KEER PER WEEK** GEBRUIKT WERD OF (BIJ BIJVOORBEELD ALCOHOL, COCAÏNE, AMFETAMINE) TWEE OPEENVOLGENDE DAGEN ZOVEEL DAT FUNCTIONEREN DE DAG ERNA BEPERKT WAS.]
C. Hoeveel dagen heeft u … gebruikt in de afgelopen 30 dagen?
[OOK VRAGEN ALS RESPONDENT BIJ VORIGE VRAAG AANGAF HET NIET VAKER DAN 3 KEER PER WEEK TE HEBBEN GEBRUIKT.]

		Jaren ooit	Aantal dagen gebruikt in afgelopen 30 dagen
A	Alcohol - elke hoeveelheid (≥ 1 glazen per dag)	__ __	__ __
B	Alcohol - ≥ 5 glazen per dag	__ __	__ __
C	Heroïne *(bruin, horse, smack)*	__ __	__ __
D	Methadon/LAAM/buprenorphine	__ __	__ __
E	Andere opiaten/analgetica *(palfium, opium, morfine, pijnstillers met opiaten)*	__ __	__ __
F	Kalmeer / slaapmiddelen *(kalmerende middelen, slaappillen) /Benzodiazepines)*	__ __	__ __

		Jaren ooit	Aantal dagen gebruikt in afgelopen 30 dagen
G	Pijnstillers	__ __	__ __
H	Antidepressiva	__ __	__ __
I	Antipsychotica	__ __	__ __
J	Snuif-cocaïne (snuifcoke, coke, C, snow, gebruik: snuiven)	__ __	__ __
K	Crack cocaïne (basecoke, wit, gebruik: basen, roken, chinezen)	__ __	__ __
L	Amfetamines (pep, speed)	__ __	__ __
M	Cannabis (hasj, marihuana, wiet, skunk)	__ __	__ __
N	Hallucinogenen (tripmiddelen, LSD, paddenstoelen)	__ __	__ __
O	Vluchtige stoffen (inhalantia, lijm, poppers)	__ __	__ __
P	Ecstasy (MDMA)	__ __	__ __
Q	GHB	__ __	__ __
R	Andere middelen, *Namelijk*____	__ __	__ __
S	Meer dan een middel per dag **combinatie van middelen behalve alcohol (elke hoeveelheid)* *Namelijk: middel + middel + middel +middel*	__ __	__ __

78. Rookt u momenteel sigaren of sigaretten?

○0 = Nee
○1 = Ja → **Hoeveel per dag (ongeveer)?**
…. sigaren / sigaretten per dag

79. Heeft u in de afgelopen 12 maanden gegokt of geld uitgegeven aan de volgende gokactiviteiten?

a.	Kansspelautomaten/fruitautomaten	O 0 Nee	O 1 Ja
b.	Casinospelen zoals roulette en blackjack	O 0 Nee	O 1 Ja
c.	Pokeren, online en/of offline	O 0 Nee	O 1 Ja
d.	Overig, b.v. bingo, krasloten, weddenschappen	O 0 Nee	O 1 Ja

BIJ 4 KEER NEE, GA NAAR VRAAG 81

80. Zo ja, hoe vaak in de afgelopen 12 maanden?
[PER ACTIVITEIT UITVRAGEN, GEBRUIK KAART 7]

	Minder dan eens per maand	1-3 keer per maand	1-4 keer per week	Dagelijks of bijna dagelijks	Niet van toepassing
a. Kansspelautomaten/ fruitautomaten	O 1	O 2	O 3	O 4	O
b. Casinospelen zoals roulette en blackjack	O 1	O 2	O 3	O 4	O
c. Pokeren, online en/of offline	O 1	O 2	O 3	O 4	O
d. Overig, b.v. bingo, krasloten, weddenschappen	O 1	O 2	O 3	O 4	O

9.3 Zorgbehoeften lichamelijke gezondheid

81. Ik wil nu graag van u weten of u op de gebieden die ik zo noem <u>op dit moment</u> hulp wilt en hulp krijgt:
A: Wilt u hulp op dit gebied?
B: Krijgt u hierbij hulp van instanties?

		**Wil hulp?**		**Krijgt hulp?**	
		Nee	Ja	Nee	Ja
**a. Lichamelijke gezondheid** *(Doorverwijzing naar arts of fysiotherapeut, advies voor ontspanningsoefeningen, eerste hulp bij letsel, hulpmiddelen)*		O 0	O 1	O 0	O 1
**b. Alcoholgebruik en gevolgen ervan** *(Onder controle krijgen van drankgebruik, voorlichting, risico's)*	O Gebruikt momenteel geen alcohol	O 0	O 1	O 0	O 1
**c. Druggebruik en gevolgen ervan** *(Onder controle krijgen van druggebruik, voorlichting, risico's op infectieziekten)*	O Gebruikt momenteel geen drugs	O 0	O 1	O 0	O 1

9.4 Psychische gezondheid

Er volgen nu een aantal vragen over uw psychische of geestelijke gezondheid en uw emotioneel welbevinden.

82. Wat vindt u van uw emotionele gezondheid, ook wel welbevinden? [GEBRUIK KAART 1]

O 1 = Vreselijk
O 2 = Slecht
O 3 = Matig
O 4 = Niet slecht/niet goed
O 5 = Redelijk
O 6 = Goed
O 7 = Prima

83. Ik noem zo een lijst op met gevoelens en gedrag van mensen. Geeft u bij elk van de uitspraken aan wat het beste uw gevoel en gedrag van de afgelopen week weergeeft. [GEBRUIK KAART 8]

	**Tijdens de afgelopen week:**	Zelden of nooit (minder dan 1 dag)	Soms of weinig (1-2 dagen)	Regelmatig (3-4 dagen)	Meestal of altijd (5-7 dagen)
1.	Stoorde ik me aan dingen, die me gewoonlijk niet storen.	O 1	O 2	O 3	O 4
2.	Had ik geen zin in eten, was mijn eetlust slecht.	O 1	O 2	O 3	O 4
3.	Bleef ik maar in de put zitten, zelfs als familie of vrienden probeerden me eruit te halen.	O 1	O 2	O 3	O 4
4.	Voelde ik me even veel waard als ieder ander.	O 1	O 2	O 3	O 4
5.	Had ik moeite mijn gedachten bij mijn bezigheden te houden.	O 1	O 2	O 3	O 4
6.	Voelde ik me gedeprimeerd.	O 1	O 2	O 3	O 4
7.	Had ik het gevoel dat alles wat ik deed me moeite kostte.	O 1	O 2	O 3	O 4
8.	Had ik goede hoop voor de toekomst.	O 1	O 2	O 3	O 4
9.	Vond ik mijn leven een mislukking.	O 1	O 2	O 3	O 4
10.	Voelde ik me bang.	O 1	O 2	O 3	O 4
11.	Sliep ik onrustig.	O 1	O 2	O 3	O 4
12.	Was ik gelukkig.	O 1	O 2	O 3	O 4
13.	Praatte ik minder dan gewoonlijk.	O 1	O 2	O 3	O 4
14.	Voelde ik me eenzaam.	O 1	O 2	O 3	O 4
15.	Waren de mensen onaardig.	O 1	O 2	O 3	O 4
16.	Had ik plezier in het leven.	O 1	O 2	O 3	O 4
17.	Had ik huilbuien.	O 1	O 2	O 3	O 4
18.	Was ik treurig.	O 1	O 2	O 3	O 4
19.	Had ik het gevoel dat mensen me niet aardig vonden.	O 1	O 2	O 3	O 4
20.	Kon ik maar niet op gang komen.	O 1	O 2	O 3	O 4

9.5 Zorgbehoeften psychische gezondheid

84. Ik wil nu graag van u weten of u op de gebieden die ik zo noem op dit moment hulp wilt en hulp krijgt:
A: Wilt u hulp op dit gebied?
B: Krijgt u hierbij hulp van instanties?

	**Wil hulp?**		**Krijgt hulp?**	
	Nee	Ja	Nee	Ja
**a. Psychische gezondheid** *(Omgaan met verdriet en tegenslag, stemmingen, uzelf mentaal sterker maken)*	O 0	O 1	O 0	O 1
**b. Weerbaarheid: voor uzelf opkomen, sterker voelen, versterken weerbaarheid en eigen kracht** *(Nee leren zeggen als u nee bedoelt, informatie over wat er in relaties mis kan gaan, zelfverdedigingcursus)*	O 0	O 1	O 0	O 1

9.6 Cognitieve vaardigheden

We willen graag weten hoe goed mensen verschillende dingen begrijpen. Daarom wil ik nu graag een korte test met u doen.

[NEEM DE HASI AF, GEBRUIK DAARVOOR DE BIJLAGE. ZET RESPONDENTNUMMER OP DE BIJLAGE. BENADRUK TIJDENS HET AFNEMEN, INDIEN NODIG, DAT SOMMIGE VRAGEN MISSCHIEN EEN BEETJE VREEMD OF SIMPEL ZIJN, MAAR DAT WE DEZE TEST BIJ ALLE MENSEN AFNEMEN EN DAT SOMMIGEN WEL MOEITE MET DEZE VRAGEN ZOUDEN KUNNEN HEBBEN.]

10 Ingrijpende gebeurtenissen

85. Ik ga een aantal gebeurtenissen noemen die mensen mee kunnen maken. Ik zou graag van u willen weten of u deze dingen ooit heeft meegemaakt en zo ja, of dat in de afgelopen drie jaar was.

		**Ooit meegemaakt?**		**Afgelopen 3 jaar meegemaakt?**	
a.	Ontslag van werk	O 0 Nee	O 1 Ja	O 0 Nee	O 1 Ja
b.	Financiële problemen (schulden, langdurig financieel niet rond kunnen komen, plotselinge achteruitgang in inkomen)	O 0 Nee	O 1 Ja	O 0 Nee	O 1 Ja
c.	Te krappe of slecht onderhouden behuizing	O 0 Nee	O 1 Ja	O 0 Nee	O 1 Ja
d.	Oneigenlijk gebruik van mijn woonruimte (dealen, illegalen, onderverhuur)	O 0 Nee	O 1 Ja	O 0 Nee	O 1 Ja
e.	Huisuitzetting	O 0 Nee	O 1 Ja	O 0 Nee	O 1 Ja
f.	Conflicten of breuken in persoonlijke relaties (denk aan (echt)scheiding)	O 0 Nee	O 1 Ja	O 0 Nee	O 1 Ja
g.	Conflict met buren en omwonenden	O 0 Nee	O 1 Ja	O 0 Nee	O 1 Ja
h.	Mishandeling [*ALLE* VORMEN VAN MISHANDELING, DOOR *ALLE* LEDEN VAN HET HUISHOUDEN]	O 0 Nee	O 1 Ja	O 0 Nee	O 1 Ja
i.	Slachtoffer van mensenhandel	O 0 Nee	O 1 Ja	O 0 Nee	O 1 Ja
j.	Overlastgevend gedrag voor anderen	O 0 Nee	O 1 Ja	O 0 Nee	O 1 Ja
k.	Sociaal isolement (weinig/geen mensen in mijn naaste omgeving om op terug te vallen)	O 0 Nee	O 1 Ja	O 0 Nee	O 1 Ja
l.	Overlijden van een gezinslid	O 0 Nee	O 1 Ja	O 0 Nee	O 1 Ja
m.	Overlijden van een naaste	O 0 Nee	O 1 Ja	O 0 Nee	O 1 Ja
n.	Een chronische ziekte van een gezinslid	O 0 Nee	O 1 Ja	O 0 Nee	O 1 Ja
o.	Een chronische ziekte van een naaste	O 0 Nee	O 1 Ja	O 0 Nee	O 1 Ja
p.	Moeite om huishouden te doen	O 0 Nee	O 1 Ja	O 0 Nee	O 1 Ja
q.	Problemen met het gebruik van alcohol	O 0 Nee	O 1 Ja	O 0 Nee	O 1 Ja
r.	Problemen met het gebruik van drugs	O 0 Nee	O 1 Ja	O 0 Nee	O 1 Ja
s.	Psychische problemen	O 0 Nee	O 1 Ja	O 0 Nee	O 1 Ja
t.	Lichamelijke gezondheidsproblemen	O 0 Nee	O 1 Ja	O 0 Nee	O 1 Ja
u.	Ontbreken van papieren en documenten (paspoort etc.)	O 0 Nee	O 1 Ja	O 0 Nee	O 1 Ja
v.	Ontslag uit algemeen of psychiatrisch ziekenhuis	O 0 Nee	O 1 Ja	O 0 Nee	O 1 Ja
w.	Contacten met politie en/of justitie	O 0 Nee	O 1 Ja	O 0 Nee	O 1 Ja
x.	(Ontslag uit) detentie	O 0 Nee	O 1 Ja	O 0 Nee	O 1 Ja
y.	Anders, namelijk: ________________________________	O 0 Nee	O 1 Ja	O 0 Nee	O 1 Ja

11 Veiligheid en geweld

11.1 Ervaren veiligheid

Nu wil ik het graag met u hebben over uw veiligheid en eventueel contact met de politie.

86. Was u in het afgelopen jaar slachtoffer van:

a.	Een gewelddadig misdrijf zoals mishandeling, verkrachting of een overval?	O 0 = Nee<br>O 1 = Ja<br>O 9 = Geen informatie
b.	Enig niet-gewelddadig misdrijf zoals inbraak, diefstal van eigendom of geld of bent u bedrogen?	O 0 = Nee<br>O 1 = Ja<br>O 9 = Geen informatie

87. Bent u in het afgelopen jaar gearresteerd of opgepakt vanwege een mogelijk misdrijf?

O 0 = Nee
O 1 = Ja
O 9 = Geen informatie

[GEBRUIK KAART 1]

	Vreselijk	Slecht	Matig	Niet slecht/ niet goed	Redelijk	Goed	Prima
**88. Wat vindt u van de veiligheid op straat, bij u in de buurt?**	O1	O2	O3	O4	O5	O6	O7
**89. Wat vindt u van de veiligheid van daar waar u woont/verblijft?**	O1	O2	O3	O4	O5	O6	O7
**90. Wat vindt u van de bescherming die u heeft tegen een beroving of aanval?**	O1	O2	O3	O4	O5	O6	O7

11.2 Zorgbehoeften veiligheid

91. Ik wil nu graag weer van u weten of u op het gebied dat ik zo noem <u>op dit moment</u> hulp wilt en hulp krijgt:
A: Wilt u hulp op dit gebied?
B: Krijgt u hierbij hulp van instanties?

	**Wil hulp?**		**Krijgt hulp?**	
	Nee	Ja	Nee	Ja
**a. Bescherming van de eigen veiligheid** *(Veiligheid in huis, op straat, in de buurt, en in relatie, bij seksualiteit en intimiteit)*	O 0	O 1	O 0	O 1

12 Zorggebruik en ervaringen met hulp

De volgende vragen gaan over de verschillende soorten hulp die u ontvangt en over uw ervaringen met hulpverlening.

12.1 Inventarisatie opvang & hulp

92. Wilt u van de volgende soorten hulp aangeven of u er ooit gebruik van heeft gemaakt en, zo ja, of u er in de afgelopen 6 maanden gebruik van heeft gemaakt?

Psychiatrische hulp	Ooit gebruikt?		Afgelopen 6 maanden?	
A Ambulante psychiatrische zorg (dagbehandeling, medicijnpoli, zorgcoördinatieteam)	O 0 Nee	O 1 Ja	O 0 Nee	O 1 Ja
B Opname in psychiatrische zorg: ziekenhuis of kliniek	O 0 Nee	O 1 Ja	O 0 Nee	O 1 Ja
C Ambulante hulp van een psycholoog of psychotherapeut	O 0 Nee	O 1 Ja	O 0 Nee	O 1 Ja
**Verslavingszorg**				
A Ambulante verslavingszorg, excl. methadonverstrekking	O 0 Nee	O 1 Ja	O 0 Nee	O 1 Ja
B Ambulante methadonverstrekking	O 0 Nee	O 1 Ja	O 0 Nee	O 1 Ja
C Opname in verslavingszorg (ziekenhuis of kliniek)	O 0 Nee	O 1 Ja	O 0 Nee	O 1 Ja
**Medische zorg**				
A Sociaal-medische spreekuren, verpleegkundigen	O 0 Nee	O 1 Ja	O 0 Nee	O 1 Ja
B Huisarts	O 0 Nee	O 1 Ja	O 0 Nee	O 1 Ja
C Algemeen ziekenhuis (incl. poli, etc.)	O 0 Nee	O 1 Ja	O 0 Nee	O 1 Ja
D Tandarts	O 0 Nee	O 1 Ja	O 0 Nee	O 1 Ja
**Overige**				
A Algemeen maatschappelijk werk	O 0 Nee	O 1 Ja	O 0 Nee	O 1 Ja
B Zelfhulpgroep *(bijvoorbeeld A.A.-groep, praatgroep van patiëntenvereniging)*	O 0 Nee	O 1 Ja	O 0 Nee	O 1 Ja
C Kredietbank	O 0 Nee	O 1 Ja	O 0 Nee	O 1 Ja
D Begeleid (zelfstandig) wonen, ambulante woonbegeleiding	O 0 Nee	O 1 Ja	O 0 Nee	O 1 Ja
E Beschermd / beschut wonen	O 0 Nee	O 1 Ja	O 0 Nee	O 1 Ja
F Jeugdhulpverlening	O 0 Nee	O 1 Ja	O 0 Nee	O 1 Ja
G Opvoedondersteuning	O 0 Nee	O 1 Ja	O 0 Nee	O 1 Ja
H Kinderbescherming	O 0 Nee	O 1 Ja	O 0 Nee	O 1 Ja
I Rechtshulp (sociaal raadslieden)	O 0 Nee	O 1 Ja	O 0 Nee	O 1 Ja
J Vrouwenhulpverlening, Stichting tegen vrouwenhandel, Steunpunt Huiselijk Geweld (ASHG)	O 0 Nee	O 1 Ja	O 0 Nee	O 1 Ja
K Politie, vreemdelingenpolitie	O 0 Nee	O 1 Ja	O 0 Nee	O 1 Ja
L GGD	O 0 Nee	O 1 Ja	O 0 Nee	O 1 Ja
O Voedselbank	O 0 Nee	O 1 Ja	O 0 Nee	O 1 Ja
P Organisatie voor werk/dagbesteding	O 0 Nee	O 1 Ja	O 0 Nee	O 1 Ja
Q Schuldhulpverlening, budgetbeheer	O 0 Nee	O 1 Ja	O 0 Nee	O 1 Ja
R Anders, namelijk ______________________________	O 0 Nee	O 1 Ja	O 0 Nee	O 1 Ja

12.2 Vertrouwen in de zorg

Ik wil u nu wat vragen stellen over uw ervaringen met hulpverlening in het algemeen.

93. Heeft u vertrouwen in de hulpverlening? [GEBRUIK KAART 3. HET GAAT HIERBIJ OM ALLE VORMEN VAN HULPVERLENING, OOK ARTSEN E.D.]

- O 1 = Helemaal niet
- O 2 = Grotendeels niet
- O 3 = Enigszins niet
- O 4 = Neutraal
- O 5 = Enigszins wel
- O 6 = Grotendeels wel
- O 7 = Helemaal wel

94. Heeft u het gevoel dat hulpverleners vooroordelen over u hebben? [GEBRUIK KAART 3]

- O 1 = Helemaal niet
- O 2 = Grotendeels niet
- O 3 = Enigszins niet
- O 4 = Neutraal
- O 5 = Enigszins wel
- O 6 = Grotendeels wel
- O 7 = Helemaal wel

95. Heeft u het gevoel dat u uw problemen beter zelf kunt oplossen, zonder de hulp van hulpverleners? [GEBRUIK KAART 3]

- O 1 = Helemaal niet
- O 2 = Grotendeels niet
- O 3 = Enigszins niet
- O 4 = Neutraal
- O 5 = Enigszins wel
- O 6 = Grotendeels wel
- O 7 = Helemaal wel

96. Heeft u het gevoel dat hulpverleners het beste met u voor hebben? [GEBRUIK KAART 3]

- O 1 = Helemaal niet
- O 2 = Grotendeels niet
- O 3 = Enigszins niet
- O 4 = Neutraal
- O 5 = Enigszins wel
- O 6 = Grotendeels wel
- O 7 = Helemaal wel

13 Algemene beoordeling

En nu weer een heel algemene vraag.

**97. Wat vindt u van uw leven in zijn geheel genomen?** [GEBRUIK KAART 1]

○ 1 = Vreselijk
○ 2 = Slecht
○ 3 = Matig
○ 4 = Niet slecht/niet goed
○ 5 = Redelijk
○ 6 = Goed
○ 7 = Prima

14 Afsluiting

Dit is het einde van de vragenlijst. Nu wil ik nog graag van u weten wat u van het interview vond.

**98. Heeft u nog vragen of opmerkingen?**

○ 0 = Nee
○ 1 = Ja, namelijk….

……
……
……
……
……
……
……
……
……

- LAAT DE RESPONDENT WETEN DAT ZIJN/HAAR MEDEWERKING ERG BELANGRIJK IS EN DAT JE HET WAARDEERT DAT HIJ/ZIJ MEE HEEFT WILLEN DOEN.

- LEG UIT DAT WE GRAAG OVER 6 MAANDEN WILLEN WETEN WAT ER MET DE RESPONDENT GEBEURD IS. VUL SAMEN MET HEM/HAAR HET **CONTACTPERSONENFORMULIER** IN.

- GEEF DE RESPONDENT DE **VERGOEDING** VOOR HET INTERVIEW EN LAAT HEM/HAAR **TEKENEN**.

- GEEF DE RESPONDENT HET **VISITEKAARTJE**.

- BEDANK DE RESPONDENT EN NEEM AFSCHEID.

Respondentnummer | | |

Contactgegevens:

Naam	Relatie tot respondent	Adres	Telefoonnummer	E-mail
**Respondent:**				

UMC St Radboud

Respondentnummer

Formulier ontvangst vergoeding

Naam:

Heeft ontvangen:

Datum:

Handtekening:

15 Vraag voor de interviewer

**99. Vul hier de eindtijd van het interview in**

_ _ UU _ _ MM

**100. Indien interview voortijdig is afgebroken, wat is daarvan de reden? Beargumenteer het voortijdig afbreken bij 'notities van de interviewer'.**

O = 1 Respondent weigerde de vragen verder te beantwoorden.
O = 2 Respondent geeft aan niet (meer) in staat te zijn verdere vragen te beantwoorden.
O = 3 Respondent beëindigde het gesprek zonder opgave van redenen.
O = 4 Respondent bleek reeds geïnterviewd.
O = 5 Vanwege dreigend/agressief gedrag van de respondent.
O = 6 Respondent was aantoonbaar onsamenhangend en onbetrouwbaar.
O = 7 Overig, namelijk
____

**101. Waren er omstandigheden die de betrouwbaarheid van de antwoorden beïnvloed kunnen hebben?**

O = 0 Nee
O = 2 Ja, namelijk:

....
....
....
....
....
....
....
....
....
....
....
....
....
....
